# Pathology and polymerase chain reaction detection of ovine progressive pneumonia (maedi) cases in slaughtered sheep in India

**DOI:** 10.14202/vetworld.2017.1401-1406

**Published:** 2017-11-30

**Authors:** Rahul Singh, Pawan Kumar, Rajendra Singh, Kuldeep Dhama, Swati Kumari, Jay Prakash Yadav, Gayatri Kashyap, Karam Pal Singh, Vidya Singh, Monalisa Sahoo

**Affiliations:** 1 Division of Pathology, ICAR-Indian Veterinary Research Institute, Bareilly, Izatnagar - 243 122, Uttar Pradesh, India; 2 Division of Veterinary Public Health, ICAR-Indian Veterinary Research Institute, Bareilly, Izatnagar - 243 122, Uttar Pradesh, India; 3 Division of Pathology, Centre for Animal Disease Research and Diagnosis, ICAR-Indian Veterinary Research Institute, Bareilly, Izatnagar - 243 122, Uttar Pradesh, India

**Keywords:** histopathology, maedi-visna, ovine progressive pneumonia, polymerase chain reaction, small ruminant lentiviruses

## Abstract

**Aim:**

The small ruminant lentiviruses are known to cause maedi-visna (MV) and caprine arthritis - encephalitis in sheep and goats, typically affecting joints, udder, lungs, and the central nervous system. The diagnosis usually involves serology, clinical signs, immunohistochemistry, and polymerase chain reaction (PCR). In the present study, the histopathologically positive pneumonia cases of MV were confirmed by PCR in lung tissue probably for the first time in India.

**Materials and Methods:**

A total of 888 lungs of adult sheep, aged between 2 and 5 years, were screened during slaughter, of which 121 were found to have pneumonic lesions. The tissues from each pneumonic lung including associated lymph nodes were collected in 10% neutral buffered formalin for histopathology. The frozen tissues of the same were also collected and stored at −20°C for PCR confirmation.

**Results:**

Three of 121 cases of pneumonic lungs of sheep revealed gross and histopathological lesions suggestive of maedi or ovine progressive pneumonia infection. These 3 cases were further confirmed by PCR technique that amplified 291-base pair DNA in the long terminal repeat sequence of MV provirus.

**Conclusion:**

This study suggests the low occurrence of MV virus (MVV) infection in India in naturally affected sheep based on pathomorphological lesions and using the molecular tool of PCR detection of the virus in tissues. Further, a combination of pathomorphology or/and PCR testing might be optimal for detecting the animals infected with MVV.

## Introduction

Small ruminant lentiviruses (SRLVs), under the *Retroviridae* family, mostly cause maedi-visna (MV) disease in sheep and caprine arthritis encephalitis disease in goats. Most of the lentiviruses infection is restricted to host, but SRLV can infect other closely related wild small ruminants, namely, red deer, roe deer, and mouflon [1,2]. MV disease is a chronic progressive and persisting infection affecting multiorgans such as lungs, mammary glands, joints, and the central nervous system [3-5]. After a prolonged incubation period, the virus causes chronic degenerative changes of smooth muscle hyperplasia (lungs), demyelination in central nervous system, indurations of udder, and proliferative synovial membrane changes (joints) [6].

Meadi-visna virus (MVV) mainly targets blood monocytes/macrophage and dendritic cell, but other cells such as epithelial cell and mammary gland also act as a reservoir of the virus and free virus in colostrum may transmit from dam to offspring. The virus becomes restricted to replication in blood monocytes but when virus through lymph node transmitted to systemic circulation it multiplies in mature tissue macrophage of the lung, mammary gland, and joint [7]. Once an animal becomes infected with MVV, the virus integrates into leukocyte DNA, and the affected animal harbors the virus for a lifetime. The burden of virus differs among individual animals; on the other hand, both asymptomatic and symptomatic animals can transmit MVV [6,8]. Maedi is diagnosed by progressive interstitial pneumonia, and visna is generally associated with meningoencephalitis. These names are Icelandic: Maedi means dyspnea (respiratory distress) and visna is equal to wasting (neurological sign); these terms arose from the early investigations of these diseases in the 1940s from Iceland. Maedi has been reported from worldwide except for Australia and New Zealand [5,9]. Transmission of the virus occurs mainly through the infected colostrums, milk from infected mother to the lamb, and/or insemination with the use of infected ram semen. Other routes of viral transmission from infected animals to healthy ones involve close contact, contaminated feed, water troughs, or milking machines [10,11]. Some reports recommended that coinfection with ovine pulmonary adenocarcinoma (OPA) amplified the transmission of MVV among sheep flocks [12].

Diagnosis of the SRLVs infection can be made clinically only in a small proportion of infected animals due to the long incubation period of diseases from months to years. Sheep may develop the clinical signs of maedi at about 3-4 years of age [3,7]. In flocks, the disease remains undetected due to the absence of obvious clinical signs. Postmortem detection of a pneumonic form of the disease rests on gross and microscopic lesions, but such lesions are not always confirmatory [13]. Difficulties in the interpretation of MV can arise in tissues with mild interstitial pneumonia, which can resemble those of lungworms or mycoplasmosis [14,15]. Pneumonia due to secondary bacterial infections usually complicates the lesions caused by MVV [16] or OPA [17].

Therefore, in such inconclusive cases, additional laboratory tests are required for confirmation of the disease. Polymerase chain reaction (PCR) assays or virus isolation are reliable and specific tests for diagnosis of this virus. These tests may be particularly useful before the animal seroconverts. MVV is usually cell-associated; the free virus is rarely found in plasma or other fluids. In living animals, viruses can be detected in leukocytes in blood or milk. At necropsy, MVV can be found in affected tissues, such as lung, mediastinal lymph node, kidney, spleen, and brain. MVV can also be detected in alveolar macrophages collected by bronchoalveolar lavage at necropsy [18,19].

This study describes the pathomorphological lesions simulating the MVV and its further confirmation by PCR. The article emphasizes that both lesions and long terminal repeat (LTR)-PCR combination might be optimal for detection of MVV infected animals.

## Materials and Methods

### Ethical approval

Due permission was taken for collection of samples from the slaughterhouse.

### Sample collection

During the period from August 2015 to April 2016, a total of 888 lungs of adult sheep were screened and 121 pneumonic lungs specimen were collected from different slaughterhouses, located in different parts of India, on the basis of gross lesions (Table-1). These animals aged mostly between 2 and 5 years. The lungs and associated lymph nodes of the affected sheep lung tissues were collected in 10% neutral buffered formalin (NBF) for histopathology and frozen tissues were stored at −20°C until further examination.

**Table-1:** Details of sample collection and diagnosis of maedi in sheep.

Place	Lungs screened	Lungs specimen collected	Histopathologically suspected for maedi	LTR-PCR Positive for Maedi-visna virus
Bareilly (Uttar Pradesh)	20	6	-	-
Gannavarum (Andhra Pradesh)	50	15	1	1
Delhi	800	90	2	2
Postmortem facility, IVRI	18	10	-	-
Total	888	121	3	3

PCR: Polymerase chain reaction, LTR: Long terminal repeat

### Histopathology

The NBF fixed tissues were cut into small pieces of 0.5 cm thickness and washed thoroughly with water for an overnight before putting in ascending grades of alcohol for dehydration. The dehydrated tissues were cleared in xylene and embedded in paraffin. Sections of 4-5 µm thickness were prepared from paraffin blocks and stained with hematoxylin and eosin (H and E), and duplicate paraffin sections were stained with Masson’s trichrome to demonstrate the smooth muscle hyperplasia in lung tissue [20]. The lesions were examined by a veterinary pathologist to give authenticity of the histopathology reports.

### Extraction of proviral MV DNA

The genomic MV proviral DNA was isolated from the frozen lung samples of histopathologically suspected animals mainly sheep using the DNeasy Kit (Qiagen, Catalog No. 69504) as per manufacturer’s protocol and stored at −20°C until used for PCR.

### Detection of MV provirus in lung sections by LTR-PCR

A PCR procedure was performed to amplify the LTR sequence of the MV provirus [18,21]. The forward primers (5-TGACACAGCAAAT GTAACCGCAAG-3, and reverse primer 5-CCA CGTTGGGCGCCAGCTGCGAGA-3) were used to amplify a 291 bp fragment of the LTR region of the MV provirus, as follows: Few modifications to the published procedure proved necessary to achieve optimum amplification. The temperature cycling procedure consisted of denaturation at 94°C for 30 s, annealing at 58°C for 30 s, and extension at 72°C for 40 s. The cycling was repeated 35 times. Each PCR reaction was initiated with a 5 min denaturation at 95°C and terminated with a 10 min extension at 72°C. All 121 lung samples were subjected to PCR using negative controls. In the negative extraction control, an equal volume of sterile deionized water was used. PCR reactions were analyzed on 1.5% agarose gels containing ethidium bromide and photographed in Gel Doc system. A sample was considered positive when the 291 bp DNA fragment of the LTR region was obtained in at least one of the replicates.

## Results

### Pathologic findings

**Figure-1:**
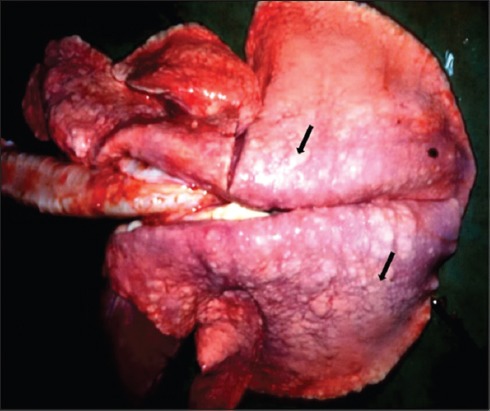
Gross appearance of maedi disease in a lung of a sheep: The affected lung is enlarged; having variable sizes (1-2 mm) whitish nodules in all the lobes (arrows) and failed to collapse.

Of the 121 pneumonic lungs samples of sheep, the maedi lesions were encountered only in 3 cases of sheep. Grossly, the affected lungs were heavier than normal, with focal or multifocal swollen nodules, and inflated. The color of the lungs was pink to white. The affected lung tissues were firm and with a somewhat meaty consistency. In two cases, rib impressions were also noticed on diaphragmatic lobes of the lungs. The lesions were found in the diaphragmatic lobes. Various numbers of pale-white granular nodules, 1-2 mm in diameter, were observed on the cut section of the lungs (Figure-1).

**Figure-2:**
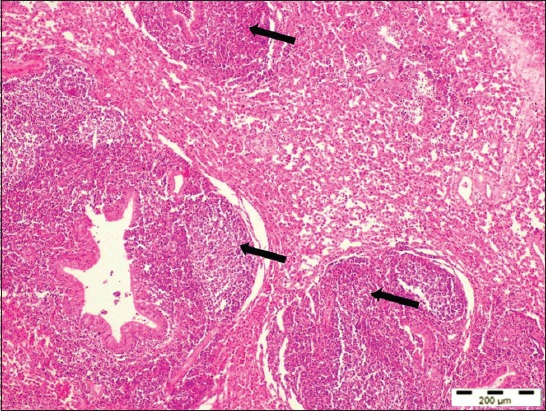
Lung section of a sheep with maedi disease showing marked lymphofollicular hyperplasia around bronchioles and blood vessels (arrows) (H and E, 40×).

**Figure-3:**
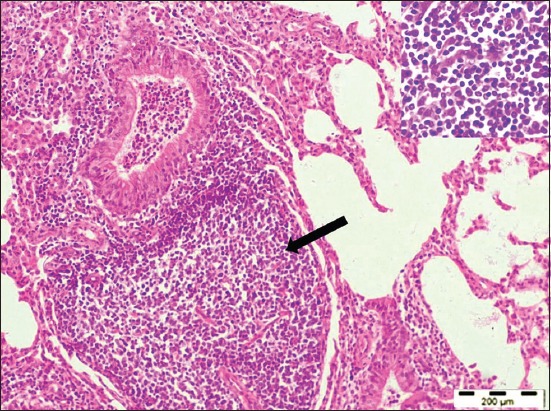
Lung section showed the presence of peribronchiolar and perivascular lymphoid follicular aggregates (arrow) with infiltration of lymphocytes (inset) into the interalveolar spaces (H and E, 100×).

**Figure-4:**
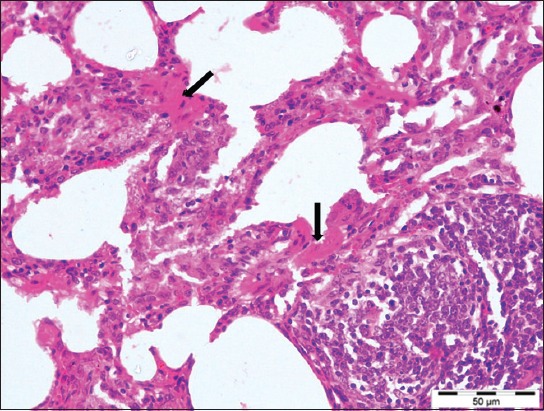
Lungs section showed characteristic smooth muscle hyperplasia of alveolar walls (arrows) with interalveolar septa thickening with mononuclear cells (H and E, 400×).

**Figure-5:**
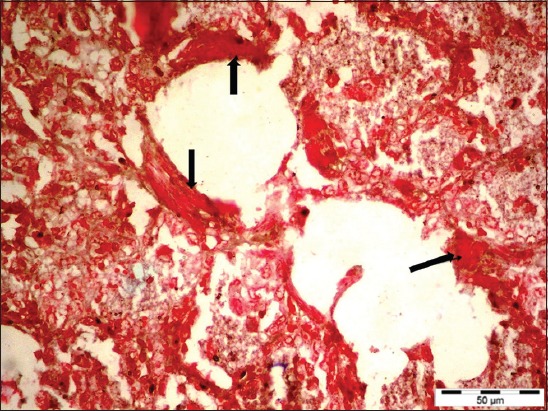
Maedi affected lung of a sheep showing characteristic alveolar smooth muscle hyperplasia (arrows) (Masson’s trichrome stain section, 400×).

**Figure-6:**
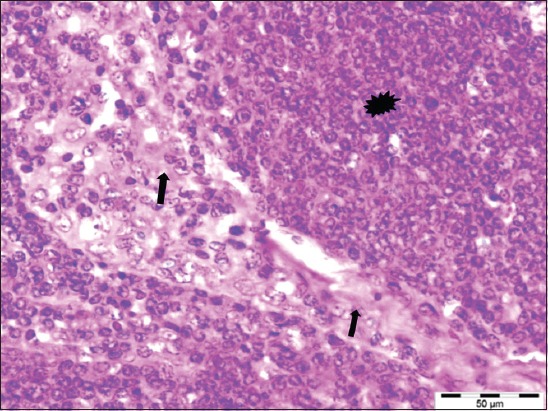
The sheep affected with maedi showing severe hyperplasia (asterisk) of the lymphocytes in the parenchyma and their infiltration into the fibrous trabecula (arrows) and the capsule of the lymph node (H and E, 400×).

Histopathologically, the lung sections showed chronic interstitial pneumonia, characterized by diffuse thickening of the interalveolar septa, mainly due to the presence of macrophages, lymphocytes, and plasma cells. Lymphocytic proliferative nodules were scattered throughout the pulmonary parenchyma primarily surrounding the bronchi, bronchioles, and blood vessels. Sometimes several foci of lymphoid hyperplasia coalesced and formed larger aggregates. The neutrophils were infiltrated in the lumen of some of the alveoli and bronchioles (Figures-2 and 3). The presence of peribronchiolar and perivascular lymphoid cells and smooth muscle hyperplasia of the alveolar wall and terminal bronchioles were the characteristic lesions observed (Figure-4). The hyperplasia of smooth muscles of the alveolar wall and the terminal bronchioles was demonstrated by Masson’s Trichome Stain (Figure-5). The mediastinal lymph nodes showed severe hyperplasia of lymphocytes with moderately expanded and some active secondary follicles with evident of germinal centers (Figure-6).

### PCR detection of MVV in the lung sections

**Figure-7:**
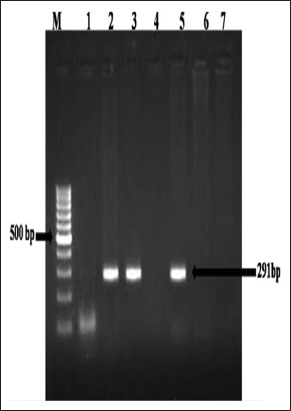
Detection of maedi proviral DNA from affected lung tissue samples: Polymerase chain reaction for long terminal repeat DNA region. Lane M: 100 bp DNA ladder, Lanes 2-3, 5: Positive amplification (291 bp), Lanes 1, 4, 6: Negative, Lane 7: Negative control.

Of 121 lung samples screened, 3 cases yielded 291 bp amplicons of LTR-DNA region of MVV by PCR (Figure-7). The same cases were also having the microscopic lesions of MV disease.

## Discussion

MV disease causes enormous production losses in small sheep and goats flocks by decreasing muscle mass and milk production [5,10,22]. Therefore, detection of such animals and their removal from the flocks is important to reduce the disease incidence. A definitive diagnosis of MVV can be made on the basis of clinical history, characteristic pathomorphological lesions, serological tests, and molecular tests [11,13,23,24].

Although MVV can infect animal at very early stage of life, MV normally does not appear in young animals except the nervous form, and in most instances, the symptoms and lesions appear in adult animals that are more than 2 years old, and that is why the detection of MV disease in young animals through macroscopic and microscopic examination alone seems to be difficult. Thus, alternative promising tests are looked for the detection of MVV. Serological prevalence of MV disease within a flock may reach up to 90%. However, the majority of infected animals do not develop the overt clinical disease [25] that is why over all conclusive idea of the frequency of occurrence of MV disease remains vague and this needs to be explored out.

In the present study, lungs and mediastinal lymph nodes of 121 sheep obtained from slaughterhouses of different regions of India were primarily screened on the basis of pathomorphology and further analyzed for the presence of proviral nucleotide sequences through an LTR-PCR method for ruling out naturally affected with meadi cases. Alvarez *et al*. [26] detected MVV seroconversion in lambs’ fed with colostrum from the seropositive ewes using LTRs and pol MVV genes-based PCR primers and found the higher sensitivity of PCR with LTR region-based primers in comparison to other tests. Serological tests were not taken into account in our study because serological based tests before seroconversion pose difficulty in confirming the diagnosis of the disease. However, PCR could be used to identify seronegative animals [13].

In our study, pulmonary lesions of MV-infected sheep were in accordance with the observation of the previous reports [11,24,27]. The dominant feature was generalized lymphoid hyperplasia with excessive peribronchiolar and discrete lymphoid follicles within lung lobules. These lymphoid nodules were frequently related to small vessels, specifically venules, even in peribronchiolar locations [21,22]. Apart from lung lesions, marked lymphoid hyperplasia and acute lymphadenitis were also observed in mediastinal lymph nodes, which were in agreement with the reported literature [28,29]. In all the three positive cases, any bacteria, mycoplasma, or chlamydia were not isolated from the infected lung tissue samples with severe involvement of maedi lesions.

The MVV has the typical genomic organization of lentiviruses which is firstly reversed transcribed into c-DNA that acts as provirus. This provirus becomes integrated to host genomic DNA through means of viral encoded integrase. Proviral DNA, having a gag, pol, and env gene, is required for virus multiplication that is flanked by LTR on 5’ and 3’ end that is why LTR-PCR may give a clear-cut picture of the virus-infected cell. Different PCR techniques have been used for MVV detection in tissue samples with different specificity and sensitivity; however, LTR-PCR method is reported to have high specificity and sensitivity [18,21,29-31]. The PCR detection of MV disease has not been carried out in India.

In the present study, three of all the screened pulmonary tissue (n=121) samples showed histopathological characteristics of MV and were also positive in LTR-PCR with amplicons size of 229 bp, which give a confirmative presence of the MV in the infected sheep samples. Thus, the LTR-PCR showed practically the same specificity and sensitivity as histopathology, in the diagnosis of this viral infection. However, our findings slightly differ with the findings of Extramiana *et al*. [18] in terms of sensitivity. These authors reported that LTR-PCR for detection of the MV provirus DNA had 100% specificity with all the screened samples, namely, blood, milk, and tissue samples of infected sheep, but differed in sensitivity, i.e. 66.7% sensitivity for milk sample and 98% sensitivity for other tissues in comparison to the two serological methods, ELISA, and AGID tests, which may be due to different viral load of tissues. Low virus load is one of the main problems for compromised PCR sensitivity for the detection of MVV [13,32]. However, the use of different PCR protocols or primers or primers from different regions (pol or gag) may be helpful for improving the sensitivity of PCR [33].

The histopathological findings in the present study are in concurrence with earlier reports [11,27,29,34]. Sasani *et al*. [23] demonstrated similar histopathological lesions of ovine lungs (26.5%) and (8.8%) with a moderate degree and severe degree of involvement, respectively, which showed the pathogen causing maedi disease (MVV) could be one of the pathogens causing chronic to subacute lymphoid interstitial pneumonia. Comparison of histopathological findings and PCR results showed that LTR-PCR is highly specific and sensitive diagnostic tool in detecting the MVV provirus in the lung samples of sheep [18,19,30]. In the present study, the frequency of occurrence of MV at abattoir was 2.47% (3 cases of 121 cases), which is akin to the previous reports that vary from 0.27% to 4.15% [24,29,35-37].

## Conclusion

The present study describes the histopathological and PCR-based detection of the advanced cases of maedi in which lymphoproliferative interstitial pneumonia and proviral DNA were detected in sheep lung samples. Pathomorphology alone poses difficulty in diagnosing those cases in which apparent lesions are not developed. Thus, a combination of pathomorphology or and PCR testing might be optimal for detecting the animals infected with MVV.

## Authors’ Contributions

RS and PK planned and accomplished the overall research work. RS, SK, and GK collected the samples and did the research works. VS and MS helped in the result analysis and drafted the manuscript. KD, KPS, and JPY revised the manuscript. All authors participated to the redaction of the manuscript. All authors have read and approved the final manuscript.
